# Arterial Blood Gas, Electrolyte and Acid-Base Values as Diagnostic and Prognostic Indicators in Equine Colic

**DOI:** 10.3390/ani13203241

**Published:** 2023-10-17

**Authors:** Luisa Viterbo, Jodie Hughes, Peter I. Milner, David Bardell

**Affiliations:** 1 North Downs Specialist Referrals, Part of Linnaeus Veterinary Limited, The Fresian Buildings 3–4, Dairy Business Park, Brewer Street, Bletchingley RH1 4QP, UK; jodie.hughes@ndsr.co.uk; 2 Department of Equine Clinical Sciences, Institute of Infection, Veterinary and Ecological Sciences, University of Liverpool, Leahurst Campus, Chester High Road, Neston CH64 7TE, UK; p.i.milner@liverpool.ac.uk (P.I.M.); david.bardell@liverpool.ac.uk (D.B.)

**Keywords:** horse, acute abdomen, gastrointestinal system, laparotomy, celiotomy

## Abstract

**Simple Summary:**

Gastrointestinal pain (colic) is one of the most frequent emergencies seen in equine practice and can be caused by several different processes, making diagnosis challenging. Deciding between medical and surgical treatment is very important and, when needed, early surgical intervention can improve survival. However, colic surgery is an invasive and expensive treatment. Results from physical examination and clinical parameters are used to identify surgical cases and establish the prognosis. No individual parameter or group of parameters has been shown to be sensitive or specific for this. This study investigated if arterial blood gas, electrolyte and acid-base analysis in conscious horses presenting with signs of colic and breathing ambient air differed from previously reported values in healthy horses and had diagnostic or prognostic value. We found significant differences between previously described data from healthy horses and the study cohort of colic cases, between some different types of colic, between surgical and non-surgical cases and between surviving and non-surviving animals. From these data, we identified specific components which demonstrated diagnostic and prognostic value.

**Abstract:**

The study aimed to investigate if arterial blood analysis in conscious horses presenting with signs of colic and breathing ambient air had diagnostic or prognostic value. Arterial blood samples from 352 horses presenting with colic at a university equine referral hospital were analysed for pH, partial pressure of carbon dioxide (PaCO_2_), partial pressure of oxygen (PaO_2_), concentrations of sodium (Na^+^), potassium (K^+^), ionised calcium (Ca^2+^) and chloride (Cl^−^), actual and standardised plasma bicarbonate concentration (HCO_3_^−^ (P) and HCO_3_^−^ (P, st)), blood and extracellular fluid base excess (Base (B) and Base (ecf)) and anion gap (AG). Results were compared to previously reported values for healthy horses, and comparisons were made between final diagnosis, treatment and survival to hospital discharge. Significant differences were found between colic cases and healthy reference values between some primary aetiologies. Overall, surgical and non-surgical colic cases differed in Ca^2+^ and Cl^−^ concentrations and Ca^2+^ differed between cases that survived to discharge and those that did not. PaO_2_ differed between small intestinal surgical cases that survived and those that did not. From these results, we developed regression models that demonstrated excellent or good predictive value in identifying the likelihood of surgical versus medical management and survival to hospital discharge.

## 1. Introduction

In horses, pain originating from the gastrointestinal tract (colic) can have different aetiologies and clinical signs, presenting both diagnostic and prognostic challenges. Causes include a spasmodic activity of intestinal smooth muscle (often caused by diet changes and parasitism), displacements, enteritis, obstructions (either simple or strangulating, the latter associated with ischemia of an intestinal segment), and dysautonomias [1]. Medical treatment is frequently successful, but in approximately 7% of cases, surgical intervention is required [2]. In these cases, early surgical treatment improves outcome and survival, reducing the likelihood of complications such as shock, ischaemia–reperfusion injury, adhesions and laminitis [3].

The decision for surgery is complex and typically based on a combination of factors such as severity of pain and response to analgesia, haemodynamic status, presence and volume of nasogastric reflux, absence of intestinal borborygmi, abnormal findings on transrectal palpation or abdominal ultrasonography, and analysis of peritoneal fluid [4,5,6,7,8,9,10,11,12,13]. Studies have also investigated peripheral venous blood lactate, fibrinogen, alcohol dehydrogenase, serum amyloid A, acid-base and electrolyte status and thromboelastography [6,14,15,16,17] as aids in clinical decision making.

Due to the serious implications of performing surgery on a horse presenting with colic and the difficulty of establishing a prognosis, several algorithms and predictive models have been developed to help clinicians achieve a timely decision [5,7,18,19]. When tested prospectively, however, both univariable and multivariable models have shown inferior performance to that predicted from the developmental cohort [20]. No single parameter, combination of tests or multivariable model has been shown to accurately identify surgical cases and give a reliable prognosis for survival and return to normal function [4,8,13]. We hypothesised that arterial blood gas, electrolyte and acid-base variables in horses displaying clinical symptoms of colic may have diagnostic and/or prognostic value. This study aimed to investigate if arterial blood gas, electrolyte and acid-base analysis, performed at the time of presentation of horses with clinical signs of colic, demonstrated diagnostic or prognostic value. 

## 2. Materials and Methods

Institutional ethical committee approval (VREC219a) and owner consent (RETH000689) was obtained for the study. Horses presenting to the Philip Leverhulme Equine Hospital, University of Liverpool, UK, for investigation and treatment of colic between June 2010 and November 2019 were enrolled in the study. Horses under 1 year of age, or where an arterial blood sample could not be obtained, were excluded. 

### 2.1. Arterial Blood Collection and Analysis

Arterial blood samples were obtained with the horse restrained using a head collar whilst standing in stocks, during initial clinical examination and prior to the administration of any medication at the equine hospital. Administration of medication before referral to the hospital could not be accurately determined for every case and, therefore, was not accounted for in the analysis. 

Samples were collected anaerobically into pre-heparinised syringes (Pico50 Arterial Blood Sampler Syringe; Radiometer Medical, Copenhagen, Denmark) via direct needle puncture of the common carotid artery by the same investigator (D.B), with the horse breathing ambient air. A volume of 1, 2 or 3 mL blood (depending on syringe volume) was collected over 2–3 breath cycles and samples were analysed immediately using an automated bench-top blood gas analyser. Temperature correction was not performed. Samples collected up to June 2014 were analysed with the ABL 77 Series Blood Gas analyser (Radiometer Medical, Copenhagen, Denmark). Automatic two-point calibration of all sensors was performed every 4 h and following installation of a new calibration solution pack. In addition, a daily external quality control was performed using commercially available tonometered reference solutions (Radiometer Qualcheck+ quality control ampoules; Radiometer Medical, Denmark). Samples collected after June 2014 were analysed with the Rapid Point 500 (Siemens, UK). Automatic one-point calibration of all sensors was performed every 30 min and two-point calibration every 2 h and following installation of a new cartridge. The measured variables were pH, partial pressure of carbon dioxide (PaCO_2_), partial pressure of oxygen (PaO_2_) and concentrations of the electrolytes sodium (Na^+^), potassium (K^+^), calcium (Ca^2+^) and chloride (Cl^−^). Actual and standardised plasma bicarbonate concentration (HCO_3_^−^ (P) and HCO_3_^−^ (P, st), respectively), blood and extracellular fluid base excess (Base (B) and Base (ecf), respectively) and anion gap (AG) were calculated by the machines from preprogramed algorithms. 

### 2.2. Acquisition of Metadata

Clinical records were examined for patient data, including age, sex, breed, body weight, diagnosis, treatment and survival to discharge from the hospital. Diagnosis was classified by location as small intestinal strangulating (SIS) or non-strangulating (SINS), large colon torsion (LCT) or non-torsion (LCNT), small colon strangulating (SCS) or non-strangulating (SCNS), viscus rupture (RUP), or other. Treatment was classified as conservative or surgical management (intestinal resection and non-resection). Diagnosis in cases which did not proceed to surgery was based on clinical data gathered at the time of admission and the final diagnosis recorded in the clinical notes. 

### 2.3. Data Analysis

Statistical analysis was performed using SPSS statistical software version 28 for Windows (IBM, Chicago, IL, USA). Measured variables were compared to a data set of results from healthy horses, previously described [21], between categories of diagnosis, treatment pathway (surgical or non-surgical) and survival, or not, to hospital discharge. 

The distribution of data for each variable was assessed using visual inspection of histograms, Q–Q plots, the Kolmogorov–Smirnov test and Levene’s test for equality of variance. Groups were compared using un-paired *t*-tests, the Mann–Whitney U test, ANOVA or Kruskal–Wallace tests dependent on distribution and variance. Bonferroni correction or Tukey’s post-hoc comparisons were performed where appropriate. Results are presented as mean (95% confidence intervals (CI)) or median (IQR). Statistical significance was assumed if *p* < 0.05. 

Comparison of arterial blood variables between horses presented with colic and healthy controls, between different classifications of colic, surgical versus non-surgical management and small intestinal strangulating versus non-strangulating lesions utilised data from all eligible cases. For analysis of variables associated with survival to hospital discharge, cases that were euthanased intra-operatively or as a result of sustaining a catastrophic injury during recovery from anaesthesia were removed from analysis.

All variables were screened for collinearity using Pearson’s *r* or Spearman’s rho, and where variables were highly correlated (>0.9), the most statistically significant or biologically plausible variable was selected. A forward likelihood ratio selection procedure was used to determine the final multivariable logistic regression models. Models developed were non-surgical management (Model 1), survival to discharge (all cases) (Model 2) and survival to discharge (small intestinal surgical cases) (Model 3). Variables with univariable *p*-values of <0.20 were entered into the models in a stepwise fashion and retained in the model if they significantly improved the fit (*p* < 0.05). The fit of the model was assessed using the Hosmer–Lemeshow goodness-of-fit test statistic. Receiver Operator Characteristic (ROC) curves were created with area under the curve (AUC) values (95% CI) calculated for variables retained in each model. Finally, each model was internally validated by calculating predictive outcome (P) using the following formula:

$$P=\frac{e^{\left(x\right)}}{{1+e}^{\left(x\right)}}$$

where *x* = *β* + (*α*_1_ × *X*_1_ + *…α_n_* × *X_n_*), *α* = coefficient of retained variable *X*, and *β* = intercept of the model.

Sensitivity, specificity, positive (PPV) and negative (NPV) predictive values were then calculated for each model from a cut-value of *p* = 0.5.

## 3. Results

Arterial blood samples were obtained from 379 horses (two hundred and twenty-four geldings, one hundred and fifty mares and five entire males) over a 9-year period. The main breeds represented were Cobs (16.1%), Thoroughbred (12.4%), Thoroughbred crosses (12.7%) and Warmblood (9.8%). Age and bodyweight distributions were 12 (1–34) years and 535.7 ± 5.8 kg, respectively (in 44 cases, no weight was recorded in the clinical records). 

Inspection of data sets showed that >98% of data were available for all variables investigated, with only small numbers of results for individual analytes not available due to machine error (PaO_2_ 1, Na^+^ 5, K^+^ 3, Ca^2+^ 3, HCO_3_^−^ (P) 3, HCO_3_^−^ (P, st) 4, Base (B) 3, Base (ecf) 3, AG 5). 

Eight horses were diagnosed with pathology not related to the gastrointestinal tract (e.g., renal mass, uterine torsion), and 19 were euthanased on putative prognostic or economic grounds following the initial assessment. These horses were removed from further analysis. Of the 352 cases classified as presenting as true colic, surgery was performed on 247, whereas 105 were managed conservatively.

Final diagnosis was small intestinal in 155 (44%), with 102 strangulating lesions and 53 non-strangulating (including seven equine dysautonomia cases); large intestinal in 151 (42.9%), comprising 30 torsions and 121 non-torsions; small colon in twelve (3.5%), six with strangulating lesions and six non-strangulating; viscus rupture in six (1.7%), and with twenty-eight (7.9%) classified as ‘other’. Intestinal resection was performed in 62 (17.6%) horses. 

Survival: 52 (14.7%) horses were euthanased either intra-operatively or following a catastrophic injury during recovery from surgery. Of the 299 horses which were managed conservatively or recovered from surgery, 252 survived to discharge, and 47 did not; in one case, survival to discharge could not be determined from clinical records. 

### 3.1. Arterial Blood Analysis for All Colics

Colics of all aetiologies were treated as a single group for comparison with healthy reference data [21]. For all analysed variables, a significant difference was found between previously reported healthy case data [21] and the colic case data; however, following Bonferroni correction, HCO_3_^−^ (P, st) and Base (B) did not remain significantly different (Table 1).

### 3.2. Comparison of Arterial Blood Gas Analysis between Categories of Colic

Significant differences in PaO_2_, K^+^, Ca^2+^, Cl^−^, HCO_3_^−^ (P), HCO_3_^−^ (P, st), Base (B), Base (ecf) and anion gap were found between a number of categories of colic. Results are summarised in Table 2. Of note, horses with small intestinal strangulating (SIS) lesions had significantly lower Ca^2+^ on admission than horses with small intestinal non-strangulating (SINS) lesions and those with large intestinal lesions (LCNT and LCT) and other categories. Chloride was also significantly lower in SIS cases compared to LCNT and other categories. Horses with small colon strangulating (SCS) lesions had significantly lower K^+^ on presentation than horses with LCNT and other categories. Horses with a ruptured viscus had significant derangements of HCO_3_^−^ (P), HCO_3_^−^ (P, st), Base (B), Base (ecf) and anion gap compared to a number of colic categories. 

### 3.3. Arterial Blood Analysis for Surgical versus Non-Surgical Colics

Calcium and Cl^−^ were significantly higher in horses undergoing non-surgical management for all colic types (Table 3). Both these variables, along with pH, were retained in Model 1 (non-surgical management) (Table 4). Due to high collinearity (>0.9) between HCO_3_^−^ (P), HCO_3_^−^ (P, st), Base (B) and Base (ecf), only HCO_3_^−^ (P) was retained as the most plausible variable for this and future regression analyses. The area under the ROC curves for pH, Ca^2+^ and Cl^−^ were 0.50 (95% CI 0.44; 0.56), 0.74 (95% CI 0.69; 0.80) and 0.66 (95% CI 0.60; 0.72), respectively. The sensitivity and specificity of Model 1 were 73% and 80%, respectively. PPV was 44.8% and NPV was 93.1%.

### 3.4. Arterial Blood Analysis for All Colics; Survival to Discharge versus Non-Survival

Fifty-two horses were excluded from the analysis due to euthanasia intra-operatively or following injury sustained during recovery from anaesthesia. Of the 299 horses that either underwent surgery and recovered from anaesthesia or were managed non-surgically and survived discharge, only Ca^2+^ was significantly different to those horses which did not survive hospital discharge (Table 5).

Calcium, along with PaO_2_ and HCO_3_^−^ (P), were retained in Model 2 (survival to discharge, all cases) (Table 6). The area under the ROC curves for PaO_2_, Ca^2+^ and HCO_3_^−^ (P) were 0.60 (95% CI 0.51; 0.69), 0.69 (95% CI 0.61; 0.77) and 0.55 (95% CI 0.45; 0.65), respectively. The sensitivity and specificity of Model 2 were 86% and 70%, respectively. PPV was 98.8% and NPV was 14.9%. 

### 3.5. Arterial Blood Analysis for Small Intestinal Lesions; Survival to Discharge versus Non-Survival

When small intestinal surgical cases that survived discharge were compared to small intestinal surgical cases that did not survive discharge, only PaO_2_ was significantly different (Table 7).

PaO_2_ and Ca^2+^ were retained in Model 3 (survival to discharge following surgical management of a small intestinal lesion) (Table 8). The area under the ROC curves for PaO_2_ and Ca^2+^ were 0.70 (95% CI 0.50; 0.74) and 0.62 (95% CI 0.60; 0.81), respectively. The sensitivity and specificity of Model 3 were 78% and 50%, respectively. PPV was 95.2% and NPV was 14.8%.

## 4. Discussion

In this study, we report the blood gas, electrolyte and acid-base status of horses presenting with signs of colic. Samples collected from the common carotid artery while the horses were breathing ambient air were analysed and compared to previously established reference intervals derived from 139 healthy adult horses at the same hospital [21]. To the authors’ knowledge, this is the first study of arterial blood gas analysis in conscious horses with colic. Significant differences were found for all variables between the healthy case data and the colic case data, most being reduced in colic horses, except for pH and anion gap, which were increased. Although HCO_3_^−^ (P, st) and Base (B) did not remain significantly different following correction for multiple comparisons, HCO_3_^−^ (P) and Base (ecf) did (Table 1). Similar to our findings, Navarro et al. [17] observed decreases in venous K^+^, pH, HCO_3_^−^ (P) and Base (B) concentrations in horses with colic when compared with horses without signs of gastrointestinal disease. Unlike our study, these authors found no difference in Na^+^, Cl^−^ or PvCO_2,_ suggesting arterial blood analysis may be a more sensitive test. Nappert and Johnson [15] also found decreases in venous HCO_3_^−^, Base (B) and K^+^ concentrations between healthy horses and horses with colic but did not find a difference in pH, AG or PvCO_2_. Both studies used venous samples collected from the jugular vein, while in our study, arterial samples were analysed. Neither study measured the partial pressure of venous oxygen (PvO_2_). In addition to this, our sample size (352 horses) was considerably larger than those investigated by Nappert et al. (50 horses) and Navarro et al. (115 horses). 

In haemodynamically stable human patients, there is a good correlation between arterial and central venous blood gas values, and venous blood can be used to estimate arterial pH and PaCO_2_. However, in a situation of shock, those estimates are not as reliable [22,23]. In our study, PaCO_2_ was lower in horses with colic, most likely suggesting a compensatory response to metabolic acidosis caused by anaerobic metabolism and lactate production, corroborated by lower values for HCO_3_^−^ and Base. This is not in agreement with a previous study using venous blood samples collected from the jugular vein [17]. Due to the variable degree of shock and severity of endotoxaemia characteristic of equine colic, arterial blood samples may be more appropriate to fully understand any acid-base disturbances present. 

Our results show that arterial blood gas analysis in isolation does not clearly discriminate for the presence of colic. Although most variables analysed were significantly different between colic cases and previously reported reference ranges in healthy horses, differences were small, with a wide variability of the data, limiting their clinical utility in this respect. This variability can be explained by individual biological variation, extent, type and/or duration of disease, transport time to the referral hospital, drugs administered prior to referral, time between sample acquisition and analysis and sample size. 

We found differences in the concentrations of a number of electrolytes between different categories of colic. Lower K^+^ concentrations in horses with colic have been previously reported when compared with healthy controls [15,16,17]. Our findings are in agreement with this, and additionally, we found K^+^ concentrations were lowest with small colon lesions and significantly lower in small colon strangulation than large colon torsion (Table 2). Several causes have been suggested for reduced K^+^ in horses with colic: reduced intake and absorption and/or excess loss caused by diarrhoea, chronic fluid therapy with lactated Ringer’s solution (which may lead to sodium-induced diuresis), metabolic alkalosis caused by the production of large volumes of gastric reflux, and administration of certain drugs [24]. It is unlikely that significant volumes of fluid therapy were administered to our study population before presentation, but this cannot be ruled out. Administration of α_2_ adrenoreceptor agonists, commonly used to facilitate examination and transport of colic horses, can also cause loss of potassium through diuresis [25]. 

Small intestinal strangulating lesions were associated with lower Ca^2+^ than non-strangulating small intestinal lesions and torsion and non-torsion of the large colon (Table 2). Significantly decreased Ca^2+^ was also found in surgical compared to non-surgical cases (Table 3) and in non-surviving horses compared to those that survived hospital discharge (Table 5). Our findings are similar to those observed by other authors [17,26,27,28]. Navarro et al. [17] reported the lowest ionised calcium concentrations in horses with ischaemic lesions, and Garcia-Lopez et al. [27] also found significantly lower preoperative Ca^2+^ (and Mg^2+^) concentrations in horses with strangulated gastrointestinal segments than in those without strangulating lesions. When measured in samples obtained preoperatively from horses with colic, serum Ca^2+^ concentration can be a consequence of diarrhoea, endotoxaemia and sepsis [28]. Hypocalcaemia is widely recognised in septic patients across species, and it has been hypothesised that it is caused by mechanisms such as alkalosis, hypomagnesaemia, chelation of calcium, altered parathyroid hormone activity, hypovitaminosis D, calcium sequestration within tissues or increases in procalcitonin [29,30,31,32]. Research in rats also suggests that the influx of Ca^2+^ from the blood to the intracellular space and secretion into the peritoneal fluid are important causes of hypocalcaemia in sepsis [33]. In light of this, it would be interesting to compare relative plasma and peritoneal fluid Ca^2+^ concentrations. Acute hypocalcaemia in the horse can manifest as ileus in animals that have some degree of gastrointestinal inflammation or sepsis or after exercise or transport [34]. Differences in Ca^2+^ concentrations were the most consistently identified disparity in our comparisons, implicating Ca^2+^ as an important component in the disease process, warranting further investigation. 

Chloride concentrations were lower in horses which underwent surgical compared to non-surgical treatment (Table 3), contradicting the findings of other studies [15,16]. Small intestinal strangulating lesions were also associated with lower Cl^−^ than large colon non-torsion colic types (Table 2). Hyperchloraemia in horses with colic signs and diarrhoea has been reported [17], whilst hypochloraemia is generally attributed to the formation of large volumes of gastric reflux [35]. This would be consistent with our findings as small intestinal strangulating lesions, which are surgical emergencies, often present with significant quantities of nasogastric reflux. 

Viscus rupture was associated with the lowest HCO_3_^−^ and Base concentrations, both these derived variables being significantly lower than small intestinal strangulating and large colon non-torsion types (Table 2), indicating severe metabolic acid-base derangements in these animals. 

To the authors’ knowledge, this study is the first to report PaO_2_ values in conscious horses with colic breathing ambient air. In colic cases PaO_2_ was significantly lower than in healthy horses: 90.1 (89.1; 91.1) mmHg and 97.5 (96.2; 98.7) mmHg, respectively (Table 1), and was the one variable which differed between horses with small intestinal surgical lesions which survived to hospital discharge (90.6 (88.5; 92.7) mm Hg) and those which did not (84.2 (80.5; 87.8) mm Hg) (Table 7). Whilst these mean values lie within the reference ranges for healthy horses, a proportion of the colic cohort would be considered borderline hypoxaemic [36]. The incidence of hypoxaemia in horses undergoing exploratory laparotomy is reported to be over six times that of horses undergoing elective surgical procedures under general anaesthesia [37,38]. This may reflect the fact that some animals are already hypoxaemic prior to surgery and may be one factor which impacts on post-operative survival. Measurement of venous oxygen tension (PvO_2_) does not compare well with PaO_2,_ and there is no consistent relationship between those two values [22], supporting the use of arterial blood gas analysis to identify higher-risk surgical candidates. 

In this study, we developed three models based on diagnosis (Model 1—non-surgical/surgical management) and outcome (Model 2—survival/non-survival to discharge for all colics, and Model 3—survival/non-survival to discharge in horses undergoing surgical management of a small intestinal lesion). Multivariable logistic regression is a powerful tool which considers the relationship between plausible variables and describes the contribution of those variables retained to the outcome studied. Models can be assessed for adequacy and thereby provide predictive values for the outcome of interest. Of those variables retained in Model 1 (Ca^2+^, Cl^−^ and pH), Ca^2+^ appeared to have the strongest influence on outcome as indicated by the adjusted odds ratio (when other predictor variables were controlled for). This was supported by its AUC of 0.74 compared to the other variables retained. Sensitivity and specificity were good for Model 1, and internal validation demonstrated that the model was excellent at predicting those horses requiring surgical management, although only fair at predicting those who can be managed medically. Ionised calcium, PaO_2_ and HCO_3_^−^ (P) were retained in Model 2, and this model demonstrated good sensitivity and specificity. It was also good at predicting horses surviving discharge following treatment; however, it was poor at predicting those horses not surviving discharge. Again, Ca^2+^ had a strong effect in this model (both from the adjusted odds ratio and an AUC of 0.69). For the final model developed (Model 3) both PaO_2_ and Ca^2+^ were retained. In this model, sensitivity was good, and PPV was excellent, although specificity and NPV were fair to poor, suggesting that Model 3 is good at correctly identifying those cases more likely to survive discharge following small intestinal surgery but not as good at predicting those that will likely not survive discharge. In Model 3, both retained variables appeared to have a similar effect on outcome. The three models developed in the present study can be used to assist clinicians and horse owners in the likely management and outcome of a horse presenting with colic in relation to specific arterial blood gas, electrolyte and acid-base parameters. In particular, it appears that Ca^2+^ is an important diagnostic and prognostic indicator in horses with colic and future studies investigating colic type may develop our observation further.

Several limitations to this study must be acknowledged. Over the 9-year period in which the samples for our study were collected, two different blood gas analysers were used to process the arterial blood samples. Both the ABL 77 Series Blood Gas analyser (Radiometer Medical, Denmark) and the Rapid Point 500 (Siemens, UK) are reliable point-of-care systems. However, despite being commonly used to analyse equine blood, neither of these machines has been validated for this purpose. Agreement between the two machines was assessed and confirmed using a limited number of samples and we therefore considered it justifiable to include data from both analysers. 

The retrospective review of clinical records would have accounted for some missing data. Additionally, information regarding the administration of medications prior to referral could not always be retrieved. This is an important limitation as we cannot discount the potential impact of drugs given before sample collection on our results. 

Horses from which arterial blood samples were collected on presentation but were diagnosed with conditions other than related to the gastrointestinal tract or were euthanised following the initial clinical examination were removed from the study. Horses which were euthanased intra- or immediately post-operatively during recovery from anaesthesia were included in comparisons between colic horses and healthy reference ranges and between colic types but were removed from the analysis of post-operative survival. The decision to euthanase horses pre- and intra-operatively could have been financially driven, or on the basis of perceived prognosis, and by removing these cases from analyses, we may have introduced a sampling bias. Additionally, our categorisation of colic types relied to some degree on presumptive rather than definitive diagnoses, which may not have been completely accurate. Small numbers of animals in some categories, notably small colon and viscus rupture, as well as the diverse range of pathologies included in our ‘other’ category, may also have influenced our findings.

## 5. Conclusions

Due to the wide range of aetiologies of equine gastrointestinal colic and the multitude of tests available to aid in its diagnosis, the decision between medical versus surgical management is rarely straightforward. Establishing a prognosis for this condition is equally challenging. Arterial blood gas, electrolyte and acid-base evaluation in horses presenting with signs of colic while breathing ambient air may supply the clinician with an additional tool for the diagnosis and prognosis of this condition. Ionised calcium particularly appears to be influenced by both the type and severity of the disease and warrants further investigation.

## Figures and Tables

**Table 1:** Univariable analysis of arterial blood gas, electrolyte and acid-base variables in 139 healthy horses and 352 horses with colic using un-paired *t*-tests for normally distributed variables and Mann–Whitney U tests * for non-normally distributed data, with significance assumed if *p* < 0.05. Variables retaining significance following Bonferroni correction are highlighted in bold. Data presented as mean (95% CI) or median (IQR) dependent on distribution. ^a^ Healthy case data set described by Hughes and Bardell [21].

	All Colic Cases *n* = 352	Healthy Cases ^a^ *n* = 139	Healthy Case Reference Intervals ^a^	*p*-Value
pH *	7.43 (7.38; 7.49)	7.42 (7.38; 7.47)	7.37–7.49	**<0.001**
PaO_2_ (mmHg) *	90.0 (76.0; 104)	97.0 (86.0; 108)	82.6–112.3	**<0.001**
**PaCO_2_ (mmHg)**	41.4 (40.8; 42.0)	45.2 (44.4; 45.9)	36.3–54.0	**<0.001**
Na^+^ (mmol L^−1^) *	134.0 (131.0; 135.0)	137.0 (135.0; 139.0)	133–141	**<0.001**
K^+^ (mmol L^−1^) *	3.28 (2.77; 3.79)	3.6 (3.2; 4.0)	3.05–4.65	**<0.001**
Ca^2+^ (mmol L^−1^) *	1.41 (1.2; 1.62)	1.54 (1.44; 1.64)	1.34–1.72	**<0.001**
Cl^−^ (mmol L^−1^) *	99.0 (93.0; 105.0)	104.0 (101.0; 107.0)	100–110	**<0.001**
HCO_3_^−^ (P) (mmol L^−1^) *	27.4 (22.0; 32.8)	28.7 (25.4; 34.1)	23.55–33.9	**<0.001**
HCO_3_^−^ (P, st) (mmol L^−1^) *	27.2 (22.8; 31.6)	28.2 (25.3; 31.1)	23.87–32.45	0.006
Base (B) (mmol L^−1^) *	3.1 (−1.8; 8.0)	4.2 (1.1; 7.3)	−0.51 to 8.8	0.004
Base (ecf) (mmol L^−1^) *	3.3 (−2.2; 8.8)	4.6 (1.2; 8.0)	−0.53 to 9.39	**0.003**
AG (mEq L^−1^) *	10.9 (4.2; 17.6)	7.2 (5.0; 9.4)	1.5–11.5	**<0.001**

**Table 2:** Comparison of arterial blood gas, electrolyte and acid-base variables between small intestinal non-strangulating (SINS) and strangulating (SIS) lesions, large colon non-torsions (LCNT) and torsions (LCT), small colon non-strangulating (SCNS) and strangulating (SCS) lesions, viscus rupture (RUP) and miscellaneous (OTHER) lesions. Analysis using ANOVA or Kruskal–Wallis * tests, with significance assumed if *p* < 0.05. Variables retaining significance following Bonferroni correction or Tukey’s post-hoc comparisons are highlighted in bold. ^†^ Adjusted *p* Value Using Bonferroni for K-W Multiple Comparisons. Data are presented as mean (95% CI) or median (IQR) dependent on distribution.

Variable	SINS *n* = 53	SIS *n* = 102	LCNT *n* = 121	LCT *n* = 30	SCNS *n* = 6	SCS *n* = 6	RUP *n* = 6	OTHER *n* = 28	*p*-Value	Tukey’s Post-Hoc Comparisons
pH	7.42 (7.41; 7.43)	7.44 (7.43; 7.45)	7.44 (7.43; 7.44)	7.43 (7.41; 7.45)	7.45 (7.39; 7.50)	7.45 (7.42; 7.49)	7.40 (7.35; 7.43)	7.43 (7.41; 7.45)	0.065	
PaCO_2_ (mm Hg)	41.9 (40.4; 42.8)	42.1 (40.8; 43.3)	41.2 (40.4; 42.1)	41.2 (38.7; 43.8)	39.8 (36.8; 42.9)	38.0 (31.3; 44.7)	36.0 (29.4; 42.6)	41.7 (39.5; 43.8)	0.177	
**PaO_2_** **(mm Hg)**	86.5 (84.3; 88.8)	90.2 (88.1; 92.2)	92.1 (90.3; 93.8)	90.4 (86.2; 94.7)	83.3 (73.4; 93.3)	87.5 (81.8; 93.2)	92.7 (85.0; 100.3)	89.4 (84.9; 93.8)	**0.032**	**SINS < LCNT (*p* = 0.017)**
Na^+^ (mmol L^−1^)	133.5 (132.6; 134.4)	133.7 (133.2; 134.2)	134.6 (133.9; 135.4)	136.0 (133.3; 138.8)	132.3 (130.0; 134.7)	135.7 (130.5; 140.9)	134.7 (133.4; 135.9)	134.9 (134.1; 135.7)	0.030	
K^+^ * **(mmol L^−1^)**	3.30 (2.37; 3.63)	3.19 (2.69; 3.69)	3.40 (2.80; 4.20)	3.40 (2.66; 4.14)	3.02 (2.20; 3.82)	2.55 (1.43; 3.67)	3.23 (2.85; 3.41)	3.42 (2.81; 4.03)	**0.003**	**SCS < LCT (*p* = 0.048) ^†^** **SCS < OTH (*p* = 0.026) ^†^**
**Ca^2+^** **(mmol L^−1^)**	1.42 (1.38; 1.46)	1.30 (1.27; 1.33)	1.44 (1.41; 1.46)	1.39 (1.34; 1.44)	1.43 (1.26; 1.61)	1.29 (1.17; 1.40)	1.37 (1.27; 1.48)	1.48 (1.44; 1.52)	**<0.001**	**SIS < SINS (*p* < 0.001)** **SIS < LCNT (*p* < 0.001)** **SIS < LCT (*p* = 0.024)** **SIS < OTH (*p* < 0.001)** **SCS < OTH (*p* = 0.039)**
**Cl^−^** **(mmol L^−1^)**	98.0 (96.6; 99.4)	97.4 (96.6; 98.3)	100.4 (99.5; 101.3)	100.5 (98.4; 102.6)	98.7 (94.6; 102.7)	97.3 (92.2; 102.5)	99.2 (93.8; 104.5)	101.0 (99.3; 102.7)	**<0.001**	**SIS < LCNT (*p* < 0.001)** **SIS < OTH (*p* = 0.017)**
**HCO_3_^−^** **(P)** **(mmol L^−1^)**	26.5 (25.5; 27.5)	28.0 (27.0; 29.0)	27.2 (26.6; 27.9)	26.8 (25.0; 28.7)	27. (23.6; 30.5)	26.3 (21.6; 30.9)	21.4 (16.3; 26.4)	27.3 (26.0; 28.6)	**0.019**	**RUP < SIS (*p* = 0.006)** **RUP < LCNT (*p* = 0.024)** **RUP < OTH (*p* = 0.042)**
**HCO_3_^−^** **(P, st)** **(mmol L^−1^)**	26.3 (25.4; 27.2)	27.9 (27.0; 28.7)	27.2 (26.7; 27.8)	26.9 (25.5; 28.4)	27.2 (23.8; 30.7)	26.8 (23.3; 30.4)	22.5 (19.1; 25.9)	27.2 (26.1; 28.3)	**0.016**	**RUP < SIS (*p* = 0.009)** **RUP < LCNT (*p* = 0.034)**
**Base** **(B)** **(mmol L^−1^)**	1.99 (0.97; 3.01)	3.68 (2.72; 4.63)	3.00 (2.38; 3.62)	2.61 (0.86; 4.35)	3.02 (−0.80; 6.83)	2.57 (−1.45; 6.58)	-2.53 (−6.87; 1.81)	2.99 (1.75; 4.23)	**0.017**	**RUP < SIS (*p* = 0.008)** **RUP < LCNT (*p* = 0.027)**
**Base** **(ecf)** **(mmol L^−1^)**	2.14 (1.02; 3.26)	3.88 (2.83; 4.94)	3.09 (2.40; 3.78)	2.56 (0.59; 4.52)	3.07 (−1.09; 7.22)	2.47 (−2.19; 7.13)	−3.18 (−8.62; 2.26)	3.12 (1.72; 4.52)	**0.016**	**RUP < SIS (*p* = 0.005)** **RUP < LCNT (*p* = 0.021)** **RUP > OTH (*p* = 0.042)**
**Anion** **Gap** **(mEq L^−1^)**	12.3 (10.9; 13.7)	11.3 (10.2; 12.5)	10.3 (9.5; 11.2)	12.2 (9.3; 15.1	9.6 (7.4; 11.8)	14.8 (10.2; 19.3)	17.6 (12.1; 23.1)	9.9 (8.3; 11.5)	**0.008**	**RUP > LCNT (*p* = 0.03)** **RUP > OTH (*p* = 0.034)**

**Table 3:** Univariable analysis for surgical and non-surgical colic cases using un-paired *t*-tests for normally distributed variables and Mann–Whitney U tests * for non-normally distributed data, with significance assumed if *p* < 0.05. Variables retaining significance following Bonferroni correction are highlighted in bold. Data are presented as mean (95% CI) or median (IQR) dependent on distribution.

	Surgical Cases *n* = 247	Non-Surgical Cases *n* = 105	*p*-Value
pH	7.43 (7.42; 7.44)	7.43 (7.43; 7.44)	0.68
PaO_2_ (mmHg)	89.4 (88.1; 90.6)	91.9 (89.9; 93.8)	0.03
PaCO_2_ (mmHg)	41.6 (40.8; 42.3)	41.0 (40.1; 42.0)	0.43
Na^+^ (mmol L^−1^)	134.0 (133.6; 134.6)	135.0 (134.3; 135.7)	0.04
K^+^ (mmol L^−1^)	3.24 (3.19; 3.3)	3.37 (3.29; 3.45)	0.02
Ca^2+^ (mmol L^−1^) *	1.37 (1.16; 1.58)	1.47 (1.30; 1.54)	**<0.001**
**Cl^−^ (mmol L^−1^)**	98.3 (97.7; 98.9)	101.0 (100.2; 101.9)	**<0.001**
HCO_3_^−^ (P) (mmol L^−1^) *	27.9 (22.4; 33.4)	26.7 (22.0; 31.4)	0.19
HCO_3_^−^ (P, st) (mmol L^−1^) *	27.5 (22.4; 32.6)	26.6 (22.6; 30.6)	0.34
Base (B) (mmol L^−1^) *	3.35 (-2.25; 8.95)	2.5 (-2.0; 7.0)	0.28
Base (ecf) (mmol L^−1^) *	3.75 (-2.25; 9.75)	2.9 (-1.9; 7.7)	0.20
AG (mEq L^−1^)	11.6 (10.9; 12.4)	10.3 (9.4; 11.1)	0.03

**Table 4:** Multivariable regression model ^a^ for non-surgical management in 352 horses presenting with colic.

Variable	Coefficient	Standard Error	Adjusted Odds Ratio	95% Confidence Interval	Likelihood Ratio *p*-Value
pH	15.41	3.97	6.7 *	(3.3; 10.1)	<0.001
Ca^2+^	8.51	1.33	4962.2	(2063.4; 67,656.6)	<0.001
Cl^−^	0.09	0.03	1.1	(1.0; 1.2)	0.02
Intercept	−136.72				

^a^ Multivariable logistic regression equation = −136.72 + [(15.41 × pH) + (8.51 × Ca^2+^) + (0.09 × Cl^−^)]. * Adjusted odds ratio log-transformed for pH.

**Table 5:** Univariable analysis of cases that survived hospital discharge and cases that did not survive discharge using un-paired *t*-tests for normally distributed variables and Mann–Whitney U tests * for non-normally distributed data, with significance assumed if *p* < 0.05. Variables retaining significance following Bonferroni correction are highlighted in bold. Data are presented as mean (95% CI) or median (IQR) dependent on distribution.

	Surviving to Discharge *n* = 252	Not Surviving to Discharge *n* = 47	*p*-Value
pH	7.43 (7.43; 7.44)	7.43 (7.41; 7.45)	0.72
PaO_2_ (mmHg)	90.6 (89.4; 91.9)	87.6 (84.4; 90.8)	0.06
PaCO_2_ (mmHg) *	42.0 (35.0; 49.0)	40.0 (32.0; 48.0)	0.18
Na^+^ (mmol L^−1^) *	134.0 (130.0; 138.0)	134.0 (129.0; 139.0)	0.16
K^+^ (mmol L^−1^)	3.31 (3.26; 3.36)	3.15 (2.99; 3.31)	0.03
**Ca^2+^ (mmol L^−1^)**	1.42 (1.4; 1.44)	1.32 (1.28; 1.37)	**<0.001**
Cl^−^ (mmol L^−1^)	99.8 (99.1; 100.3)	97.7 (95.8; 99.5)	0.01
HCO_3_^−^ (P) (mmol L^−1^)	27.6 (27.2; 28.1)	26.7 (25.1; 28.3)	0.27
HCO_3_^−^ (P, st) (mmol L^−1^)	27.4 (27.0; 27.8)	26.9 (25.6; 28.2)	0.43
Base (B) (mmol L^−1^)	3.24 (2.79; 3.69)	2.47 (0.91; 4.03)	0.42
Base (ecf) (mmol L^−1^)	3.43 (2.94; 3.92)	2.47 (0.72; 4.22)	0.36
AG (mEq L^−1^)	10.5 (9.9; 11.1)	12.7 (10.6; 14.8)	0.04

**Table 6:** Multivariable regression model ^a^ for survival to discharge for all colics in 299 horses presenting with colic.

Variable	Coefficient	Standard Error	Adjusted Odds Ratio	95% Confidence Interval	Likelihood Ratio *p*-Value
PaO_2_	0.06	0.02	1.1	(1.0; 1.1)	0.003
Ca^2+^	5.90	1.27	365.3	(30.5; 4372.5)	<0.001
HCO_3_^−^ (P)	0.15	0.04	1.2	(1.1; 1.3)	<0.001
Intercept	−15.45	3.41			

^a^ Multivariable logistic regression equation = −15.45 + [(0.06 × PaO_2_) + (5.90 × Ca^2+^) + (0.15 × HCO_3_^−^(P))].

**Table 7:** Univariable analysis of horses with small intestinal surgical lesions which survived hospital discharge and those which did not, using un-paired *t*-tests for normally distributed variables and Mann–Whitney U tests * for non-normally distributed data, with significance assumed if *p* < 0.05. Variables retaining significance following Bonferroni correction are highlighted in bold. Data presented as mean (95% CI) or median (IQR) dependent on distribution.

	Survival to Discharge *n* = 84	Non-Survival to Discharge *n* = 27	*p*-Value
pH	7.43 (7.42; 7.44)	7.44 (7.42; 7.45)	0.73
**PaO_2_ (mmHg)**	90.6 (88.5; 92.7)	84.2 (80.5; 87.8)	**0.003**
PaCO_2_ (mmHg)	42.7 (41.5; 43.9)	42.2 (40.1; 44.4)	0.71
Na^+^ (mmol L^−1^) *	134.0 (131.0; 137.0)	134.5 (128.5; 140.5)	0.52
K^+^ (mmol L^−1^)	3.22 (3.12; 3.31)	3.09 (2.91; 3.26)	0.17
Ca^2+^ (mmol L^−1^)	1.35 (1.32; 1.38)	1.3 (1.24; 1.35)	0.07
Cl^−^ (mmol L^−1^)	97.7 (96.7; 98.7)	96.6 (94.8; 98.3)	0.27
HCO_3_^−^ (P) (mmol L^−1^)	28.0 (27.1; 29.0)	28.0 (26.0; 29.9)	0.96
HCO_3_^−^ (P, st) (mmol L^−1^)	27.7 (26.9; 28.6)	27.8 (26.1; 29.4)	0.92
Base (B) (mmol L^−1^)	3.56 (2.62; 4.51)	3.6 (1.8; 5.4)	0.97
Base (ecf) (mmol L^−1^)	3.83 (2.8; 4.84)	3.83 (1.85; 5.79)	1.0
AG (mEq L^−1^)	11.3 (10.2; 12.5)	11.2 (9.1; 13.2)	0.89

**Table 8:** Multivariable regression model ^a^ for survival to discharge of 110 horses undergoing surgical management of a small intestinal lesion.

Variable	Coefficient	Standard Error	Adjusted Odds Ratio	95% Confidence Interval	Likelihood Ratio *p*-Value
PaO_2_	0.08	0.03	1.1	(1.0; 1.1)	0.049
Ca^2+^	3.26	1.66	26.2	(1.0; 670.6)	0.005
Intercept	−9.70	3.44			

^a^ Multivariable logistic regression equation = −9.70 + [(0.08 × PaO_2_) + (3.26 × Ca^2+^)].

## Data Availability

Not applicable.
